# Physiological and transcriptomic comparisons shed light on the cold stress response mechanisms of *Dendrobium* spp

**DOI:** 10.1186/s12870-024-04903-1

**Published:** 2024-04-01

**Authors:** Zhiyuan Li, Shunjiao Lu, Shuangshuang Yi, Shunjin Mo, Xiaoyun Yu, Junmei Yin, Changqing Zhang

**Affiliations:** 1 Sanya Institute of China Agricultural University, Sanya, Hainan, 572025 China; 2 Tropical Crops Genetic Resources Institute, Key Laboratory of Crop Gene Resources and Germplasm Enhancement in Southern China, Chines Academy of Tropical Agricultural Sciences, Danzhou, Hainan, 571737 China; 3 Department of Ornamental Horticulture, College of Horticulture, China Agricultural University, 100193, Beijing, China; 4 Hainan Engineering Center of Tropical Ornamental Plant Germplasm Innovation and Utilization, 571737, Danzhou, Hainan China; 5 Sanya Research Institute, Chinese Academy of Tropical Agricultural Sciences, 571101, Sanya, China

**Keywords:** *Dendrobium* spp, Cold stress, Signaling pathway, WGCNA, Transcriptomic patterns

## Abstract

**Background:**

*Dendrobium* spp. comprise a group of tropical orchids with ornamental and medicinal value. *Dendrobium* spp. are sensitive to low temperature, and the underlying cold response regulatory mechanisms in this group are unclear. To understand how these plants respond to cold stress, we compared the transcriptomic responses of the cold-tolerant cultivar ‘Hongxing’ (HX) and the cold-sensitive cultivar ‘Sonia Hiasakul’ (SH) to cold stress.

**Results:**

Chemometric results showed that the physiological response of SH in the later stages of cold stress is similar to that of HX throughout the cold treatment. Orthogonal partial least squares discriminant analysis (OPLS–DA) revealed that soluble protein content and peroxidase activity are key physiological parameters for assessing the cold tolerance of these two *Dendrobium* spp. cultivars. Additionally, weighted gene co-expression network analysis (WGCNA) results showed that many cold response genes and metabolic pathways significantly associated with the physiological indices were enriched in the 12 detected modules. The Kyoto Encyclopedia of Genes and Genomes (KEGG) and gene ontology (GO) enrichment analyses of the 105 hub genes showed that *Dendrobium* spp. adapt to cold stress by regulating signal transduction, phytohormones, transcription factors, protein translation and modification, functional proteins, biosynthesis and metabolism, cell structure, light, and the circadian clock. Hub genes of the cold stress response network included the remorin gene *pp34*, the abscisic acid signaling pathway-related genes *PROTEIN PHOSPATASE 2 C* (*PP2C*), *SNF1-RELATED PROTEIN KINASE 2* (*SnRK2*), *ABRE-BINDING FACTOR 1* (*ABF1*) and *SKI-INTERACTING PROTEIN 17* (*SKIP17*), the Ca^2+^ signaling-related GTP diphosphokinase gene *CRSH1*, the carbohydrate-related gene *STARCH SYNTHASE 2* (*SS2*), the cell wall biosynthesis gene *CINNAMYL ALCOHOL DEHYDROGENASE* (*CAD7*), and the endocytosis-related gene *VACUOLAR PROTEIN SORTING-ASSOCIATED PROTEIN 52 A* (*VPS52A*).

**Conclusions:**

The cold-responsive genes and metabolic pathways of *Dendrobium* spp. revealed in this study provide important insight to enable the genetic enhancement of cold tolerance in *Dendrobium* spp., and to facilitate cold tolerance breeding in related plants.

**Supplementary Information:**

The online version contains supplementary material available at 10.1186/s12870-024-04903-1.

## Background

*Dendrobium* is one of the largest genera of tropical orchids. These economically important plants are widely distributed in tropical and subtropical Asia and eastern Australia [1]. Importantly, interest in *Dendrobium* spp. species is broad, such as in traditional medicine, specialty cosmetic materials, and ornamental horticulture [2–4]. In China and some subtropical regions, *Dendrobium* spp. cultivation history and breeding research are relatively recent. Notably, *Dendrobium* spp. flower supply primarily originates from Thailand or Taiwan, where the overall temperature is suitable for their growth. However, in the majority of subtropical regions, uncertain cold waves cause low temperature damage to *Dendrobium* spp. plants, including gradual leaf yellowing, slower growth, and decreased flower longevity [5]. To adapt to cold weather, plants have evolved a range of coping strategies at the molecular and physiological levels [6]. These adaptation strategies must be identified, optimized, and analyzed to improve plant cold tolerance and maintain crop productivity in the future. Hence, phenotypic adjustments and changes in the *Dendrobium* spp. physiological, biochemical, molecular, and genetic information are needed to improve their cold tolerance.

In general, temperatures 0–15℃ and below 0℃ are considered triggers of chilling and freezing stress, respectively [7]. Plants exposed to cold utilize antioxidant enzyme systems, osmotic regulation, and cell membrane structure alterations to launch defense mechanisms and accumulate physiological metabolites to reduce cold-induced damage [8]. The biosynthesis of plant physiological metabolites is controlled by a complex regulatory network of cold stress-related genes, and plant cold tolerance is a quantitative trait regulated by multiple genes [9]. In rice (*Oryza sativa*), CHILLING-TOLERANCE DIVERGENCE 1 (COLD1) is a thermoreceptor that senses cold signals and induces calcium influx [6]. Cold signaling in plants relies on multiple molecular messengers and transcription factors (TFs) to initiate signal transduction pathways and to regulate downstream cold-regulated genes (CORs). CORs stimulate the coordinated activation of multiple biochemical pathways and encode a range of proteins of known functions. For instance, these proteins play roles in reactive oxygen species (ROS) detoxification, carbohydrate biosynthesis and cellular structures [10–12]. CORs bind to mitogen-activated protein kinases (MAPKs) and Ca^2+^-dependent protein kinases (CDPKs) to phosphorylate INDUCER OF *CBF* EXPRESSION 1 (ICE1) and activate cold stress–related pathways [13]. Meanwhile, Ca^2+^ influx is closely related to ROS signaling, which directly regulates MAPK cascades through the activation of ROS or abscisic acid (ABA) [14, 15]. The C-repeat (CRT)-binding factor (CBF)-dependent pathway and the ABA signaling pathway are the two key pathways activated in response to low temperature [16]. The ABA–responsive element-binding factors (ABFs)/ABA-responsive element-binding proteins (AREBs) are bZIP TFs that regulate plant cold tolerance in an ABA–dependent manner [17]. Moreover, CBFs/DEHYDRATION-RESPONSIVE ELEMENT BINDING (DREB) proteins, the most important cold response regulators, are strongly induced by ICE1 and trigger *COR* gene expression to combat cold stress [18].

Given that plants experience cold stress in winter, *Dendrobium* spp. cultivars with high cold tolerance in China and subtropical regions are needed. However, there are many drawbacks to conventional *Dendrobium* spp. hybrid breeding methods, such as high breeding cost, time-consuming nature, and low efficiency. Therefore, genetic modification is a convenient option to enhance *Dendrobium* spp. low temperature tolerance [19]. Nonetheless, information on the genes and molecular mechanisms regulating *Dendrobium* spp. cold responses remains limited. Previously, 196 *Dendrobium* spp. cultivars were selected for cold tolerance studies, and the relevant physicochemical indices were determined and analyzed. Among the 196 cultivars, *Dendrobium* spp. cultivar ‘Hongxing’ exhibited superior cold tolerance.

To characterize the low temperature response of *Dendrobium* spp., we evaluated the differences between a cold–sensitive cultivar (‘Sonia Hiasakul’) and a cold–tolerant cultivar (‘Hongxing’) in terms of physiology and transcriptomic changes. First, the phenotypes of both *Dendrobium* spp. cultivars in response to cold (10℃) were characterized. Then, their key physiological parameters were analyzed by chemometrics. Finally, the upstream regulatory genes and metabolic pathways associated with *Dendrobium* spp. cold responses were identified by weighted gene co–expression network analysis (WGCNA). This study provides essential data to explore the molecular mechanisms of cold stress and to facilitate the breeding of cold–resistant *Dendrobium* spp. cultivars.

## Results

### Physiological responses of *Dendrobium* spp. leaves to cold stress

**Fig. 1:**
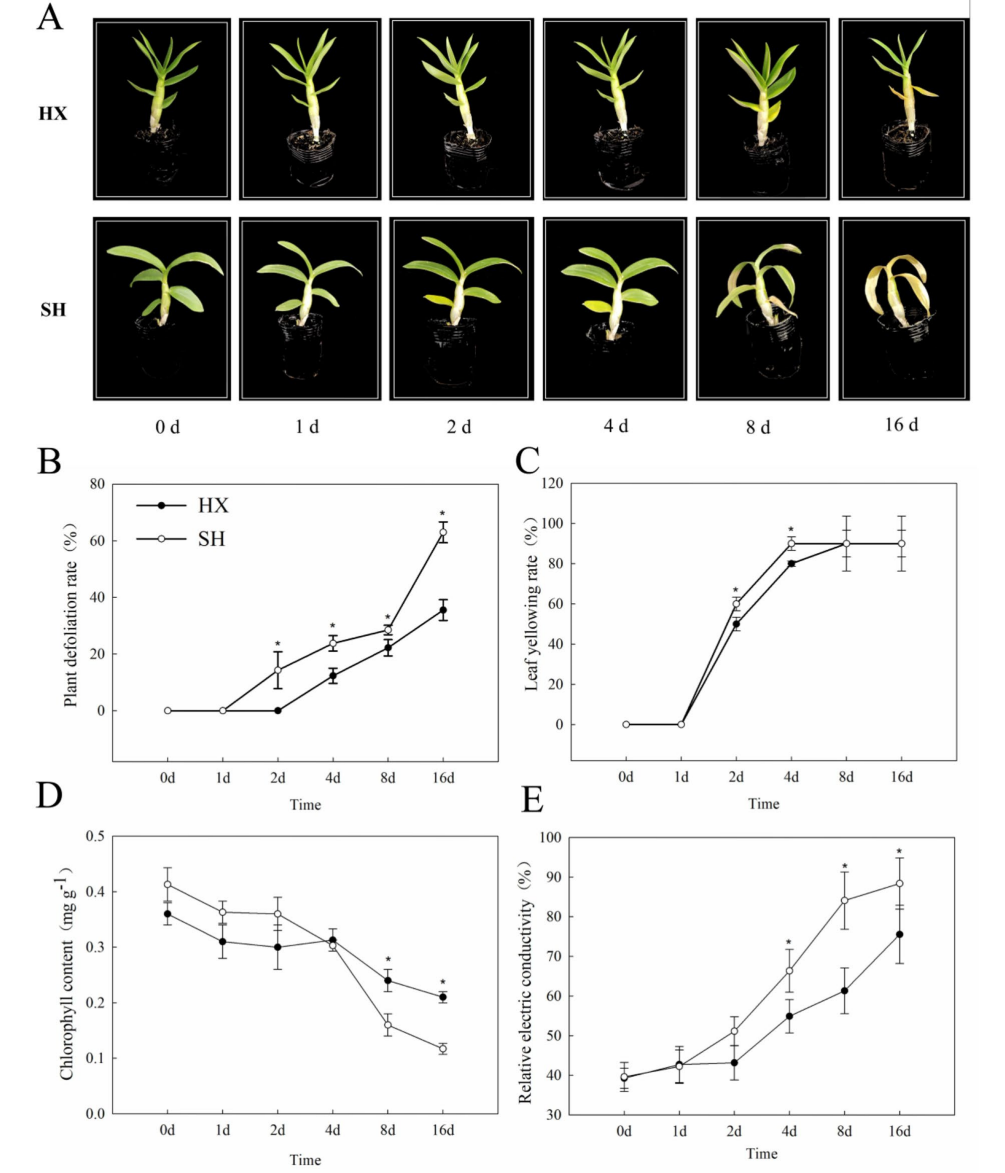
Morphological changes of *Dendrobium* spp plants under 10℃ cold stress. (**A**) The *Dendrobium* spp phenotypes. HX, the cold–tolerant genotype ‘Hongxing’; SH, the cold–sensitive genotype “Sonia Hiasakul”; the scale bar is 10 cm. (**B**) Plant defoliation and (**C**) Leaf yelllow rate, *n* = 10. (**D**) Chlorophyll content and (**E**) Relative electric conductivity, *n* = 3. Error bars represent standard deviation. “*” represent a significant differences based on one–way ANOVA followed by Tukey’s multiple comparison (*P* < 0.05)

In this study, two *Dendrobium* spp. cultivars, HX and SH, were tested and analyzed (Fig. 1A). The morphology of the two cultivars was significantly altered by cold stress (10 °C). SH leaves did not change significantly after exposure to cold stress for 1 d, while old leaves gradually turned yellow after 2 d of cold treatment; this trend continued from 4 to 16 d. In contrast, HX leaves did not substantially change after 4 d of cold treatment, but slowly turned yellow at 8 d. The defoliation rate of HX was lower than that of SH after 2 d of cold treatment (*P* < 0.05, Fig. 1B). However, the leaf yellowing rate of HX was lower than that of SH at 2–4 d of cold treatment (*P* < 0.05, Fig. 1C). The leaf chlorophyll content of HX was higher than that of SH after an 8-d cold treatment (*P* < 0.05), while the relative electrical conductivity (REC) of HX was consistently lower than that of SH after 4 d of cold treatment (*P* < 0.05, Fig. 1D and E). The leaf phenotypes of the *Dendrobium* spp. under cold stress confirmed that HX and SH represent cold–tolerant and cold–sensitive genotypes, respectively.

**Fig. 2:**
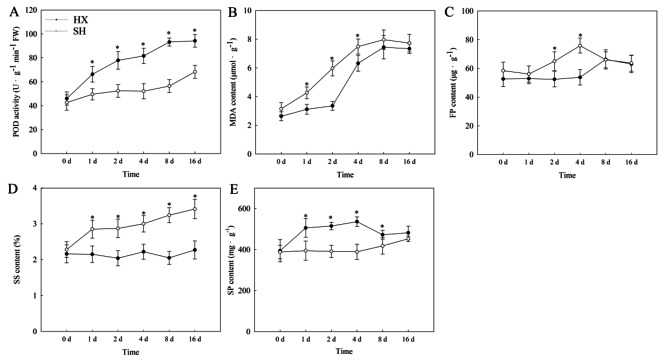
Analysis of physiological indices of HX and SH under 10℃ cold stress. (**A**) Peroxidase (POD) activity; (**B**) Malondialdehyde (MDA) content; (**C**) Free proline (FP) content; (**D**) Soluble protein (SP) content; (**E**) Soluble sugar (SS) content. Error bars represent standard deviation (*n* = 3). “*” represent a significant differences based on one–way ANOVA followed by Tukey’s multiple comparison (*P* < 0.05)

To assess the physiological responses of the two *Dendrobium* spp. cultivars under cold treatment, the peroxidase (POD) activity and the malonaldehyde (MDA), free proline (FP), soluble protein (SP) and soluble sugar (SS) contents in leaves were evaluated (Fig. 2). The POD activity of HX leaves was higher than that of SH throughout the cold treatment, except for the control (0 d) (*P* < 0.05, Fig. 2A). The POD activity of SH leaves increased by 60.1% after 16 d of cold treatment compared to control leaves. In addition, compared to SH samples at 0 d, the POD activity of HX leaves increased after 1 d of cold treatment. POD activity in HX leaves increased by 42.3% at 2 d, 67.8% at 4 d, 95.0% at 8 d, and 97.3% at 16 d compared to the control (*P* < 0.05).

The MDA content of SH leaves increased after 1 d of cold treatment, while HX accumulated substantial MDA levels after 2 d of cold treatment (*P* < 0.05, Fig. 2B). Notably, MDA levels in HX leaves were consistently lower than those in SH throughout the 16-d cold treatment (*P* < 0.05). The FP content in both SH and HX leaves significantly increased after 2 and 4 d of cold stress (29.8% and 13.4%, respectively) compared to the control (Fig. 2C).

The SS content of SH leaves was much higher than that of HX leaves. Compared to the control leaves, the SS content in SH leaves significantly increased after 1 d of cold treatment (*P* < 0.05). However, the SS content of HX leaves did not change throughout the cold stress period (Fig. 2D). The SP content of SH leaves peaked after 16 d of cold stress (a 16.6% increase compared to the control). The SP content of HX leaves peaked after 4 d of cold treatment, followed by a significant decrease of 11.3% after 16 d (Fig. 2E).

### Chemometric analysis of the physiological data

**Fig. 3:**
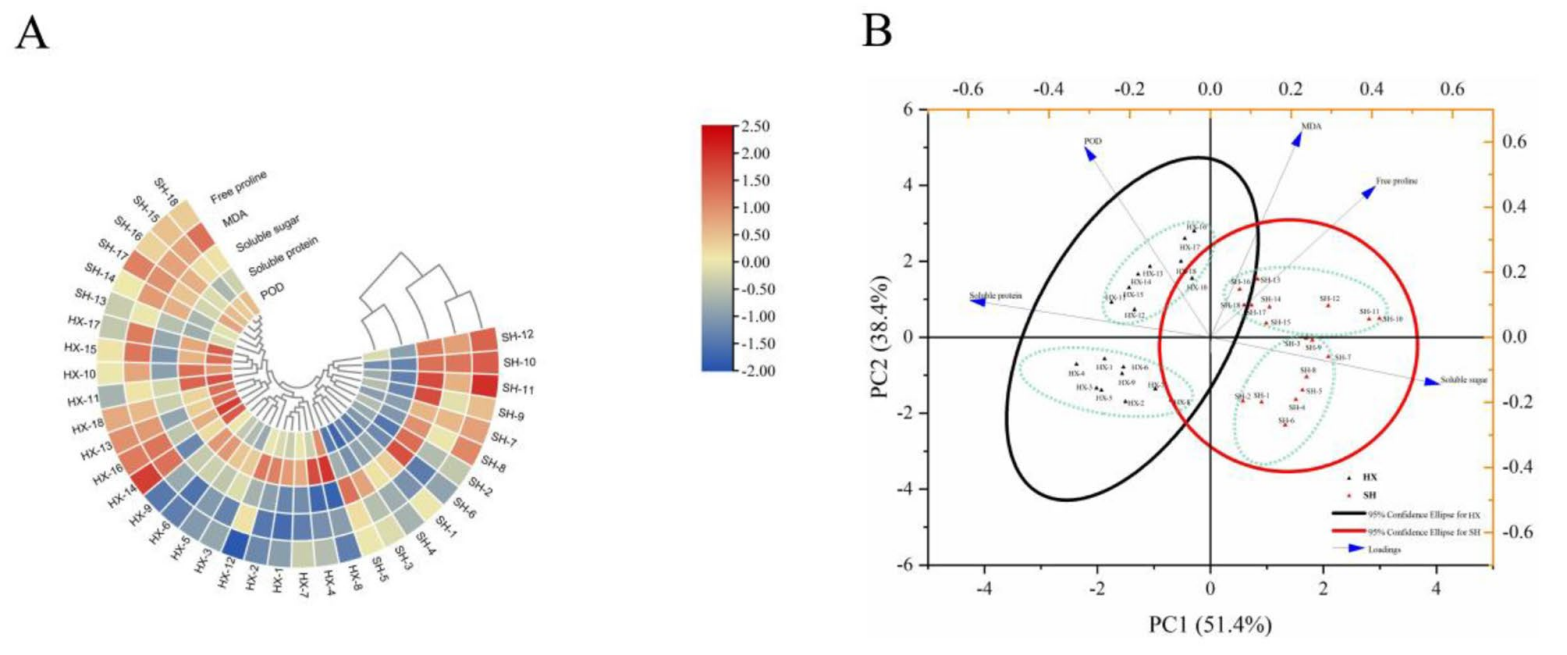
The cluster heat map (CHM) (**A**) and principle component analysis (PCA) (**B**) of the physiological indices. The numbers 1–3, 4–6, 7–9, 10–12, 13–15 and 16–18 represent three replicates after cold treatment 0, 1, 2, 4, 8 and 16 d, respectively

The five physiological indices of the two *Dendrobium* spp. cultivars were assessed for normal distributions and further analyzed based on chemometrics (Fig. S1). The cluster of physiological indices of the two cultivars under cold treatment was analyzed according to the cluster heatmap (CHM) method (Fig. 3A). The 36 samples were divided into two major clusters: HX 1–18 (all cold treatments) and SH 13–18 (8-d and 16-d cold treatments) in the first cluster, and SH 1–12 (0-d, 1-d, and 2-d cold treatments) in the second cluster.

Principal component analysis (PCA) was used to analyze and describe the grouping characteristics of the physiological metrics of the two *Dendrobium* spp. cultivars. The total variance of PC1 was 51.4%, and the sample separation was obvious. HX 1–18 represented negative values (black triangle), and SH 1–18 represented positive values (red triangle) (Fig. 3B). In PC2 (38.4%), the two cultivars were classified into two groups (HX 1–9 and HX 10–18 in HX samples, and SH 1–9 and SH 10–18 in SH samples). PC1 and PC2 were used to distinguish the differences between the cultivars and the cold stress treatments, respectively. Furthermore, the MDA, FP, and SS contents of PC1 exhibited positive loadings, whereas SP content and POD activity showed negative loadings. PC2 had positive loadings from SP, POD, MDA and FP, while SS showed negative loadings.

**Fig. 4:**
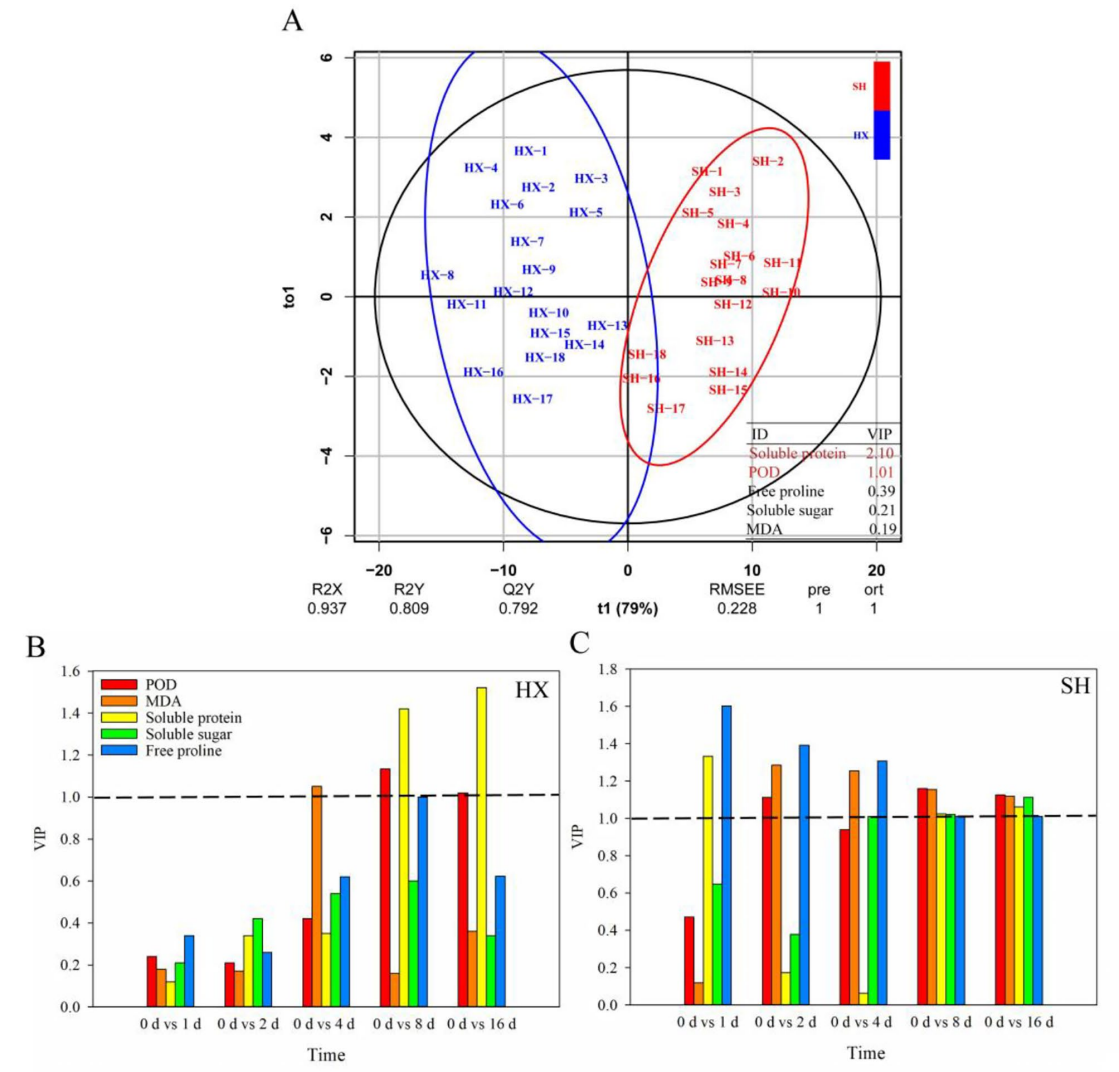
The orthogonal partial least squares discriminant analysis (OPLS–DA) of physiological indices. (**A**) Blue and red lines indicate the distribution of the physiological data in HX and SH, respectively; (**B** and **C**) Black dashed lines represent the key nodes of VIP = 1. The numbers 1–3, 4–6, 7–9, 10–12, 13–15 and 16–18 represent three replicates after cold treatment 0, 1, 2, 4, 8 and 16 d, respectively

Furthermore, the key physiological indices of the two *Dendrobium* spp. cultivars under cold stress were evaluated using Orthogonal Partial Least Squares Discriminant Analysis (OPLS–DA). A VIP threshold greater than 1.0 was used as the screening criterion for key physiological indices. SP (VIP = 2.10) and POD (VIP = 1.01) were identified as important physiological indices to evaluate cold tolerance in the two cultivars (Fig. 4A). In addition, SP (8–16 d), POD (8–16 d) and MDA (2 d) were the important physiological indices in HX (Fig. 4B), while SP (1 d, 8–16 d), SS (4–16 d), POD (1 d, 8–16 d), MDA (2–16 d), and FP (1–4 d) were important in SH (Fig. 4C).

### Identifying genes associated with the *Dendrobium* spp. cold response

Based on the phenotypic and physiological responses of the two *Dendrobium* spp. cultivars, we performed transcriptome sequencing of leaves at 0, 4, and 8 d of cold stress. Sequencing libraries generated 59,865,094–82,252,042 clean reads from 18 *Dendrobium* spp. samples (nine cold–sensitive genotypes and nine cold–tolerant genotypes), with greater than 95% of both Q20 and Q30 bases. The proportion of mapped reads in each sample library was above 76.95% (Table S2). Ideal within and intergroup correlation coefficients were obtained for all samples, and the cumulative score of PC1 (44.21%) and PC2 (14.94%) was 59.15% in the PCA (Fig. S2A and B).

**Fig. 5:**
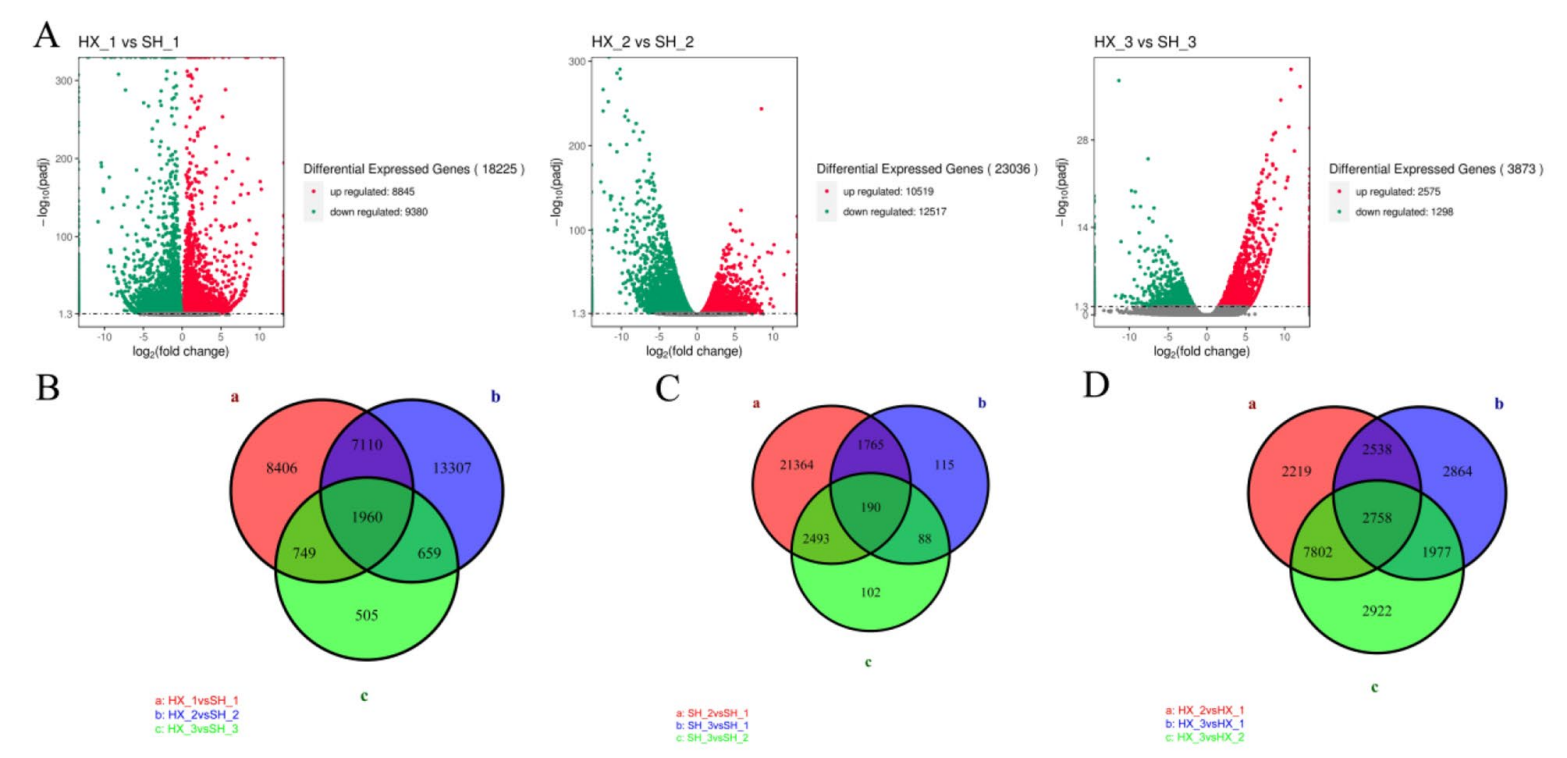
Differential and common expression genes in ‘Hongxing’ (HX) and ‘Sonia Hiasakul’ (SH). (**A**) Volcano plot analysis between two *Dendrobium* spp cultivars; (**B**) Analysis of shared genes of HX; (**C**) Analysis of common genes of SH; (**D**) Analysis of common genes between HX and SH. HX_1 vs SH_1, HX_2 vs SH_2 and HX_3 vs. SH_3 indicates the comparison between HX and SH at 0, 4 and 8 d, respectively

To identify differentially expressed genes (DEGs) related to cold treatment in *Dendrobium* spp., we compared the Fragments Per Kilobase of transcript per Million mapped reads (FPKM) values per gene at different time points. The screening criteria used to retain DEGs in the different groups were |log_2_FC| ≥ 2 and false discovery rate (FDR) < 0.05 [20]. We obtained 18,225, 23,036, and 3873 DEGs at 0, 4, and 8 d of cold treatment, respectively. The number of co-expressed genes was higher at 0 d and 4 d; this number decreased substantially at 8 d (Fig. 5A). A total of 1,960 co–expressed genes was detected in the two *Dendrobium* spp. cultivars at the three time points (Fig. 5B). Overall, 2,758 shared genes were detected in HX under cold stress, while only 190 were found in SH (Fig. 5C and D). These data indicate that the genes and metabolic pathways associated with *Dendrobium* spp. cold stress responses are complex and diverse.

### Construction of the cold response gene co–expression network

**Fig. 6:**
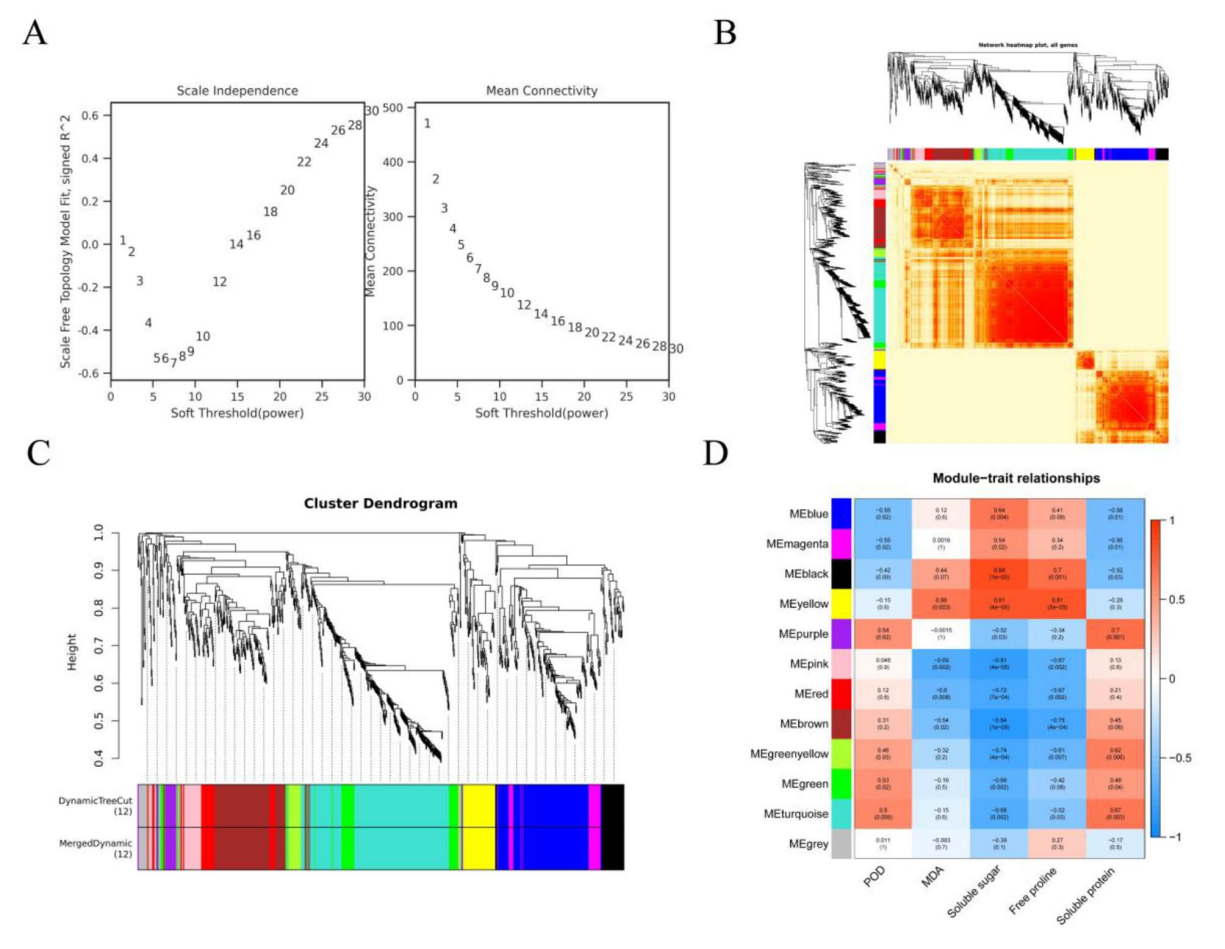
Weighted gene co–expression network analysis (WGCNA) of genes related to cold responses in *Dendrobium* spp. (**A**) Soft power plot. The abscissa represents the soft threshold (β), the ordinate represents the scale–free topology model fit index (left), and the mean connectivity index (right); (**B**) Cluster heatmap of all genes in the 11 modules are shown; (**C**) Clustering dendrogram of genes and module division. The dynamic tree cut represents the module divided according to the expression of each gene, while the merged dynamic is the result of merging similar modules according to the dynamic tree cut; (**D**) Correlations between modules and traits (time). Each row corresponds to a module, and each module cell contains the corresponding correlation and *p*-value

To identify candidate genes associated with *Dendrobium* spp. cold tolerance, a co-expression network was constructed by combining WGCNA and physiological indices in response to cold stress. The 1,960 co–expressed genes were optimized, and 12 modules containing 896 candidate genes were detected (Fig. 6B and C). Trait correlation analysis among the different physiological indices and modules revealed that the pink, red, and brown modules had significant negative correlations with the physiological indices, while the remaining eight modules also reached significant differences from the physiological indices (*P* < 0.05, Fig. 6D). Therefore, the candidate genes from the 12 modules were further screened for key cold tolerance genes in *Dendrobium* spp.

### Detecting hub genes in the 12 modules

We identified 105 high-connectivity hub genes associated with the cold stress response pathway in the 12 modules. Functional annotation of the co–expression network showed that the cold response involves nine components, including signal transduction, phytohormones, transcription factors, protein translation and modification, functional proteins, biosynthesis and metabolism, cell structure, light, and the circadian clock (Fig. S3).

Based on the 105 selected hub genes, 31 (29.52%) are related to signal transduction pathways, including 11 genes encoding serine/threonine kinases (AT2G23950, AT5G45780, Ca^2+^ signaling-related GTP diphosphokinase gene (*RCH1*), AT5G61350, *HHIX1*, *PBL23*, *D6PKL1*, AT4G20940, *14–3–3*, *ZRK7*, AT5G10290, and *CBL*), two genes encoding MAPK signaling proteins (*MAPK11* and *CAD7*), five genes encoding Ca^2+^ signaling–related proteins (*ATP1*, *CDPK8*, *CLCC*, *PMT* and *HOX20*), two calmodulin-related genes (*PH1* and *CRSH1*), one gene encoding a remorin (*pp34*), and ten other genes involved in diverse signal transduction pathways. Among these 31 genes, the connectivity values of *RCH1* (41.92), AT5G61350 (23.76), *D6PKL1* (38.14), *14–3–3* (25.20), *ZRK7* (33.62), *CAD7* (42.45), *CLCC* (49.94), *HOX20* (29.27), *CRSH1* (108.17), and *pp34* (25.21) were relatively high, indicating that serine/threonine kinases, MAPK signaling, Ca^2+^ signaling, and calmodulin may modulate *Dendrobium* spp. cold tolerance (Table S3).

A total of 14 genes (13.33%) from the phytohormone category was identified from 105 hub genes. According to the KEGG annotation results, *PP2C* (TRINITY_DN45082_c0_g3 and TRINITY_DN46981_c0_g2), *SnRK2*, and *ABF1* in the ‘plant hormone and signal transduction–abscisic acid (ABA)’ metabolic pathway (ko04075) are key players in the *Dendrobium* spp. cold stress response. In particular, *PP2C* (107.63) had high connectivity, suggesting that it may be the hub gene governing *Dendrobium* spp. cold tolerance. In addition, we identified three ABA-induced genes, namely *SKIP17*, *TSPO*, and *BLH1*, with connectivity values of 42.88, 2.15 and 2.94, respectively. Finally, two ethylene–responsive genes (*ERF1B* and *RAP2–12*), one auxin–responsive gene (*AUX1*), and four jasmonic acid–related genes (*CAD7*, *JAR1*, *MYC2* and *ILR1*) were identified. Among these genes, *CAD7* (42.45) and *JAR1* (29.96) had high connectivity values and may also represent hub genes in the *Dendrobium* spp. cold stress response (Tables S3 and S4).

Genes involved in carbohydrate biosynthesis may improve plant cold tolerance [9]. Gene annotation results identified 18 genes related to biosynthesis and metabolism. Eight of these 18 genes (*SS2*, *SPS4*, *FRU*, *UDP73E1*, *UDP6*, *SWEET14*, *bZIP11*, and *DP6*) are involved in ‘starch and sucrose metabolism’ (ko00500). Three genes (*CoIs*, *UDP7*, and *UDP73E1*) were enriched in ‘carbon metabolism’ (ko01200), along with five (*AMT2*, *CAD7*, *CCoAOMT*, *TTL*, and *TTL* [2]) in ‘phenylpropanoid biosynthesis’ (ko00940), and two (*PEPC* and *PEPC* [2]) in glycolysis (or gluconeogenesis) (ko00010) (Tables S3 and S4).

Plant cellular structures (including the cell wall, membranes, organelles, and endocytosis) change upon sensing low temperatures. *CSA8*, *GRPS*, *GPAT*, AT4G06744, and *CSB6* were detected in the ‘cell wall–related’ genes. Among the three ‘membrane–related’ genes, *UPF0591* is annotated as a peroxisomal membrane protein, and *NIP* is an aquaporin. *BAX4* was identified in the cysteine–rich transmembrane module. In addition, we selected five ‘organelle’ (GO:0043226)–related genes, namely *RAP2–12*, *TSPO*, *BLH1*, *SKIP17*, and *PUB12*. We also observed that endocytosis-related genes (*ERD4*, *ERD4* [2], *HSP70*, and *VPS52A*) are involved in the plant cold stress response. Notably, *VPS52A* had a high connectivity value (85.66) (Tables S3 and S4).

We also found that genes related to light and the circadian clock were induced by cold stress. *FTSH*, *ELP1*, *DLC1*, *PG1*, *LHC–II*, *LDSH6* and *RGA3* were detected in the ‘light’ terms. Among the circadian clock-related genes, only *ZTL* was related to the cold stress response (Table S3). In addition, transcription factor families such as MYB (*MYB58*), NAC (*NAC68* and *NAC53*) and bHLH (*bHLH3*, *bHLH42*, *bHLH51*, *bHLH104*, *bHLH124* and *bHLH143*) were closely associated with *Dendrobium* spp in response to cold stress.

### Expression analysis of hub genes

The expression patterns of core genes with higher connectivity values in the *Dendrobium* spp. cold stress response were analyzed. We focused on the expression patterns of nine genes related to ABA signaling, calmodulin, remorin, carbohydrate and cell structure. Upregulation of *PP2C*, *SnRK2*, *ABF1*, *CRSH1*, *pp34*, *CAD7* and *VPS52A* and downregulation of *SS2* and *SKIP17* may promote *Dendrobium* spp. cold tolerance.

**Fig. 7:**
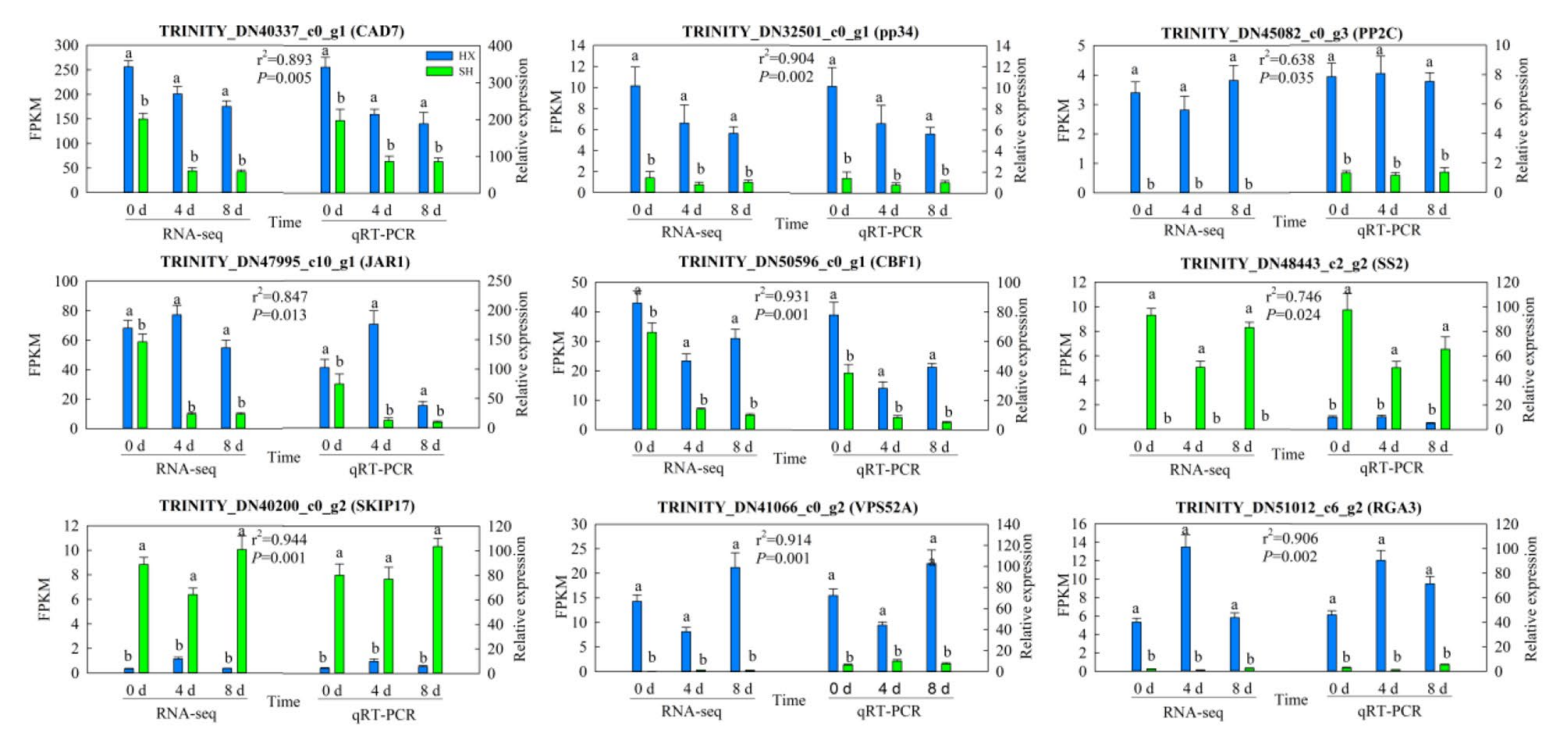
qRT-PCR analysis of 9 cold responsive genes between cold sensitive and cold tolerant genotypes. Error bars represent standard deviation (*n* = 3). Significant differences between the two cultivars were based on one–way ANOVA followed by Tukey’s multiple comparison (*P* < 0.05). The Pearson correlation coefficient is expressed as r^2^. CAD7, cinnamyl alcohol dehydrogenase 7; pp34, remorin pp34 protein; PP2C, protein phosphatase 2 C; JAR1, jasmonic acid-amido synthetase 1; CBF1, C-repeat binding factors 1; SS2, starch synthase 2; SKIP17, Ski-interacting protein 17; VPS52A, vacuolar protein sorting-associated protein 52 A; RGA3, disease resistance protein RGA3

To verify the reproducibility of the RNA–seq data, we analyzed the expression patterns of nine hub genes from two cultivars in 12 modules by reverse transcription quantitative PCR (RT-qPCR). The correlation coefficients of all nine hub genes were between 0.6 and 1.0, indicating that their expression patterns were similar to those obtained through RNA–seq, and further confirming the reliability of the cold-responsive genes detected through WGCNA (Fig. 7).

## Discussion

### Comparing the physiological changes of HX and SH under cold treatment

Plants undergo a series of physiological changes under stress conditions to maintain cellular homeostasis and stabilize growth and development. Antioxidant enzymes (POD), membrane lipid peroxidation indicators (MDA), and osmotic adjustment compounds (including FP, SP, and SS) regulate cellular homeostasis in plants under cold stress [5]. Nevertheless, the response mechanisms are not identical across different plant cultivars. Changes in POD activity affect antioxidant responses and modulate upstream signaling and signal transduction pathways [21]. POD activity and SP content in HX were higher than those in SH from 0 to 16 d of cold stress. HX may harbor more metabolic compounds related to cellular structures compared to SH, allowing it to easily adapt to temperature changes over time [9]. In the CHM analysis, SH leaves exposed to cold stress for 8–16 d were grouped into the same category as HX leaves exposed to cold stress for 0–16 d. The metabolic changes observed in SH samples from 8 to 16 d of cold stress were similar to the changes evident in HX throughout the 16-d cold stress treatment, during which the cold defense system had been initiated. Because the cold response in SH was initiated after cold treatment, it took longer for stress-related physiological compounds to accumulate [7, 9]. These results suggest that more physiological metabolites accumulated in HX leaves than in SH throughout the cold treatment, providing greater protection against cold damage [9].

OPLS–DA revealed that HX and SH exhibit different degrees of physiological responses under cold treatment. In this study, the main physiological parameters involved in regulating HX cold tolerance were POD, MDA and SP. As an important antioxidant enzyme, POD participates in ROS detoxification, limits oxidative stress, and can slow damage to the plant body [18]. MDA is the final decomposition product of membrane lipid peroxidation, and its content reflects plant damage status [22]. HX leaves accumulated more MDA than SH leaves, which exhibited mild lipid peroxidation. This difference may be due to the stronger antioxidant system in HX, possessing higher ROS scavenging efficiency under cold stress. Some cellular osmotic regulators, including FP, SS, and SP, can modulate cell penetration potential, reduce cell freezing points, and enhance plant chilling tolerance [18]. Plants with strong cold tolerance exhibit greater SS levels in response to cold stress, which limits ROS accumulation [9, 23]. The critical physiological indicators of cold stress in the SH cultivar are SS and FP. These results suggest that SH may limit ROS levels through sugar accumulation to mitigate the adverse effects of cold stress, while FP would maintain cellular osmotic potential to resist cold invasion. Consistent with our findings, a previous study demonstrated that differential osmotic metabolite levels (including SS and FP) in *Zanthoxylum bungeanum* leaves caused the differences between cold–sensitive and cold–tolerant cultivars [9]. These results indicate that cold–tolerant herbs and woody plants behave similarly in response to cold stress, both adapting through powerful antioxidant systems and the accumulation of suitable osmotica.

### Gene expression analysis and hub gene identification under cold stress

Many genes were differentially expressed in the two *Dendrobium* spp. cultivars after cold stress compared to the control (0 d). The respective gene expression patterns of the two cultivars differed greatly in response to cold stress, suggesting that the two genotypes activated two distinct sets of genes in response to cold [9]. Candidate gene functions can be identified by comparative analysis of DEG patterns between cold–sensitive and cold–tolerant cultivars. In this study, a scale–free co–expression network was constructed using the co–expressed genes of the two *Dendrobium* spp. genotypes, and module and hub genes associated with its cold responses were identified.

When plants are exposed to cold, a series of signal transduction pathways are activated to trigger stress responses and promote survival [24]. Calcium ions are important second messengers for sensing external signals and mediating signal transduction in response to abiotic stress [25]. Calmodulin–like proteins (CaMLs) and calmodulin–binding proteins (CBLs) are calcium sensors that improve plant stress tolerance [26, 27]. In this study, cold stress triggered the GTP diphosphokinase *CRSH1*, a core gene involved in Ca^2+^ signaling [28], which plays a critical role in enhancing low temperature tolerance in *Dendrobium* spp. Remorin is a plasma membrane (PM)/lipid raft–associated protein that promotes cell signaling, PM transport, and phytohormone responses by acting directly with signaling proteins such as receptor kinases [29]. We identified a remorin protein with high connectivity, pp34, as significantly upregulated by cold stress. This result suggests that pp34 may affect *Dendrobium* spp. cold tolerance, a finding that is consistent with the results of a study on *Zanthoxylum bungeanum* [9]. However, the role of remorin proteins in protecting plants against cold stress remains unclear, requiring further validation by biochemical assays [30, 31].

Self–regulation of the expression patterns of phytohormone signaling pathways is another adaptive strategy to protect plants in cold environments. Cold signals accelerate the massive accumulation of ABA accumulation, which alters the metabolic processes of cells and enhances plant cold tolerance [32]. ABA signaling play an important role in establishing cold tolerance in various plants, including Arabidopsis, rice, and tomato [33–35]. Stress triggers PYL–ABA–PP2C complex formation: the ABA receptors (PYLs) interact with PP2C phosphatases, which activates SnRK2 kinase activity and stimulates downstream gene expression to establish a stress response [36]. In this study, the WGCNA revealed that the *PP2C* and *SnRK2* hub genes were upregulated in guard cells under cold stress (Fig. S4A). SKIP regulates core ABA signaling genes, including *ABF*s and *PP2C*s, which improves Arabidopsis abiotic stress tolerance [37]. The observed cold-induced downregulation of *SKIP17* may be responsible for the reduced cold sensitivity of *Dendrobium* spp. Furthermore, we identified the hub gene *ABF1* in the co–expression network [38]. The identified hub genes included components involved in each step of ABA signaling, indicating that ABA signaling is likely a key means to improve cold tolerance in *Dendrobium* spp. The response of peach (*Prunus persica*) [39] and eggplant (*Solanum melongena*) [40] to cold signals indicates that the hormone signal transduction pathway regulate the cold response pattern in plant, and there are significant differences between the cold-sensitive and cold-tolerant genotypes. Therefore, manipulating ABA signaling represents a potential strategy to improve *Dendrobium* spp. cold tolerance.

Plants use carbohydrates as their main energy source to regulate normal physiological activities, growth, and development. Moreover, carbohydrates play an essential role in controlling plant secondary metabolism and stress responses [41]. In general, increased carbohydrate–related enzyme activity contributes to enhanced plant survival in cold conditions [42, 43]. For example, starch biosynthesis or degrading enzymes (such as SS2) can regulate plant growth, development, and metabolic status, in addition to affecting plant sensitivity to stress [9]. In this study, carbohydrate metabolic pathways and specific enzymes, including starch and sucrose metabolism, glycolysis, a raffinose biosynthesis enzyme, and a UDP–glucosyltransferase enzyme, were identified as regulators of the cold stress response. Carbohydrate metabolism promotes *Dendrobium* spp. cold tolerance, as it reduces the freezing point of cell fluid and protects the protein membrane from cold-induced damage. Many differentially expressed genes related to carbohydrate metabolic pathways have been identified by KEGG enrichment analysis in two cold–sensitive and cold–tolerant eggplant cultivars [40], further indicating that carbohydrate metabolism is closely linked to plant cold tolerance.

Low temperature mainly damages cell membranes and causes water loss. These changes can further lead to cell metabolism disorders, and even cell death [44]. Plants have gradually evolved adaptive strategies to mitigate cellular structural damage during long–term cold adaptation. Cell wall formation depends on the mutual coordination of cell structures and slows plant cell damage [45]. Lignin, which fills in the cellulose framework of plants, is an important cell wall constituent and plays a role in supporting and protecting plant cell wall biosynthesis [46]. In this study, the cinnamyl–alcohol dehydrogenase gene *CAD7* and the caffeoyl–CoA O–methyltransferase gene *CCoAOMT*, two genes related to lignin biosynthesis, were detected in the KEGG enrichment analysis [47, 48]. These data suggest that cold signals induce the expression of lignin biosynthesis–related genes, likely to enhance cell wall biosynthesis, slow cellular structure damage, and promote *Dendrobium* spp. adaptation to a cold environment. Upstream of the metabolic changes triggered by cold stress, cold receptors in hardy plants sense low temperature signals and trigger a series of adaptive responses. These responses include activation of calcium signaling pathways, such as those downstream of the cold receptor COLD1 reported in rice [6, 49]. Plant cell walls perceive external cold signals earlier than organelles and membranes. Although the cell wall does not directly participate in signal transduction, structural disturbances or distortions that occur under adverse environmental conditions indirectly affect the transmission of chemical signals, typically leading to calcium influx. Therefore, the cell wall may play a cold receptor–like role in plants.

Plant cells maintain PM integrity and stress tolerance by balancing endocytosis and exocytosis [50]. The endocytic efficiency of the PM depends on the cooperation of two pathways: clathrin–mediated endocytosis (CME) and clathrin–independent endocytosis (CIE) [51]. CME is the most widely studied and prevalent transcytosis pathway. This pathway rapidly senses environmental changes and cooperates with CIE to turn over membrane proteins by regulating the balance between endocytosis and exocytosis, which impacts plant abiotic stress tolerance [52]. CME-associated clathrin–coated vesicles entering cells form early endosomes and receive endocytic material through the endosomes to complete sorting [53]. A portion of the cargo proteins are used to recycle the PM. Another fraction of the cargo proteins is processed and ubiquitinated, eventually transporting the vesicles to late endosomes and lysosomes [54]. In the present study, we identified four endocytosis- and exocytosis–related candidate genes affecting *Dendrobium* spp. cold tolerance (Fig. S4B). Among these candidate genes, *ERD4* and *ERD4* [2] are sparn proteins that regulate CME. *HSP70* is a vesicle peeling–related gene, and VPS52A is a vacuolar sorting–related protein [55–57]. *VPS52A* had the highest connectivity as a hub gene in the co–expression network, suggesting that VPS52A-centred endocytosis likely positively regulates *Dendrobium* spp cold tolerance.

In cold conditions, some plants accelerate flowering (vernalization) or seed germination, while other plants acquire cold tolerance (low-temperature domestication) [58]. We identified the light-responsive ATP-dependent zinc metalloprotease *FTSH* gene and clock-associated PAS gene *ZTL*, and suggest that they are independently regulated by light and low temperature. Highly-expressed FTSH is involved in the cold acclimation of alfalfa (*Medicago sativa*) chloroplasts and may function as a photoreceptor in low temperature-mediated transcriptional regulation [59]. Constitutive *ZTL* expression elongates hypocotyls and delays flowering in Arabidopsis [60]. In this study, both *FTSH* and *ZTL* showed high connectivity; we hypothesize that low temperature may regulate the light responses and biological clock of *Dendrobium* spp., leading to changes in flowering.

Transcription factors plays key roles in stress response and tolerance by regulating the expression of stress-related genes in plants [7]. There are several transcription factors (*NAC58/39* and *MYB75*) verified to be involved in cold tolerance of *Dendrobium* orchids in previous study [61, 62]. These transcription factors belong to the homologue genes of *NAC68/53* and *MYB58* respectively identified in our co-expression network, which further supports the critical role of the NAC and MYB transcription factor families in improving the low temperature tolerance of *Dendrobium* spp.

**Fig. 8:**
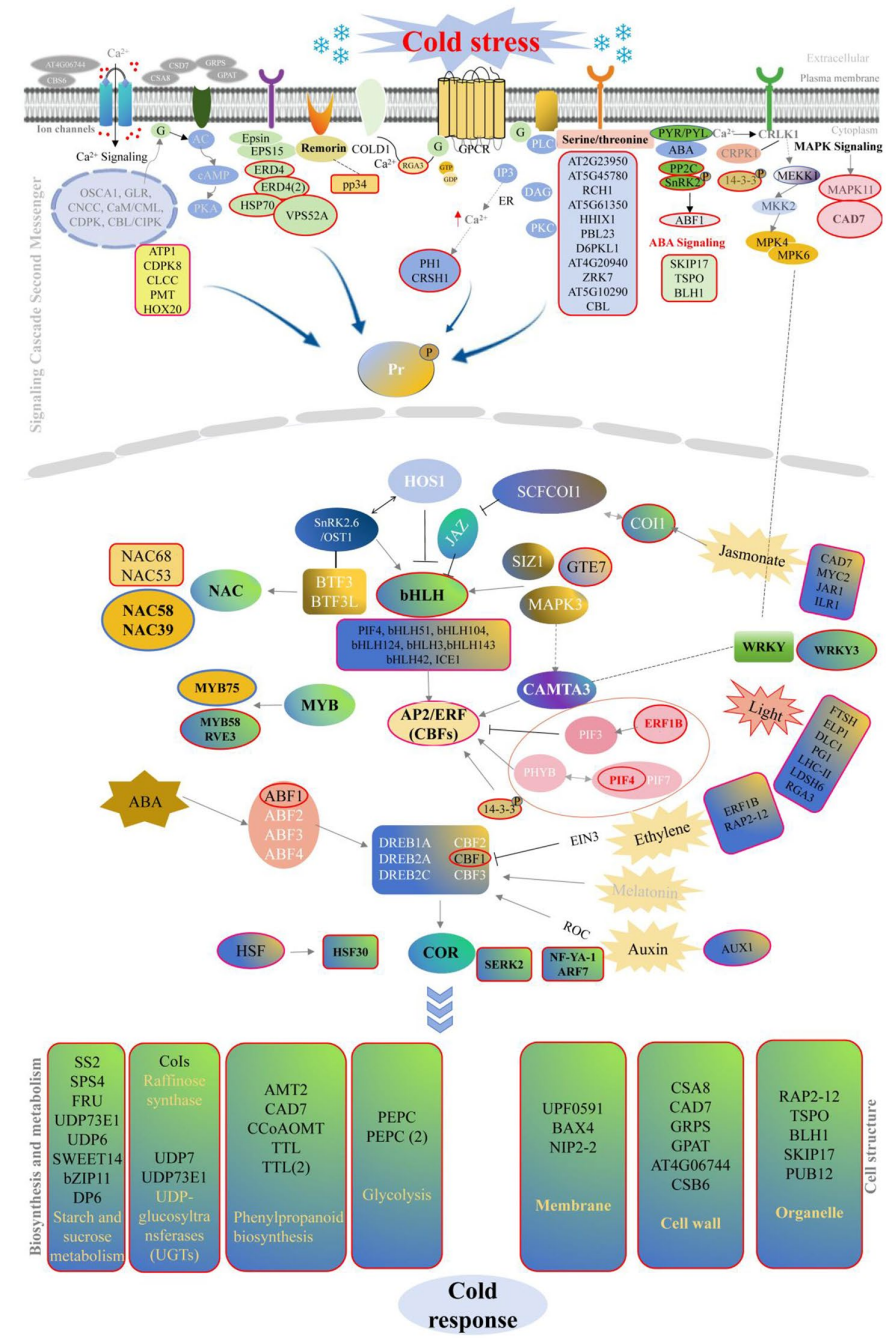
Putative module and cold response pathways in *Dendrobium* spp. Red boxes indicate cold response factors identified from the co–expression network, while blue boxes represent the validated genes involved in regulating cold tolerance of *Dendrobium* species

**Fig. 9:**
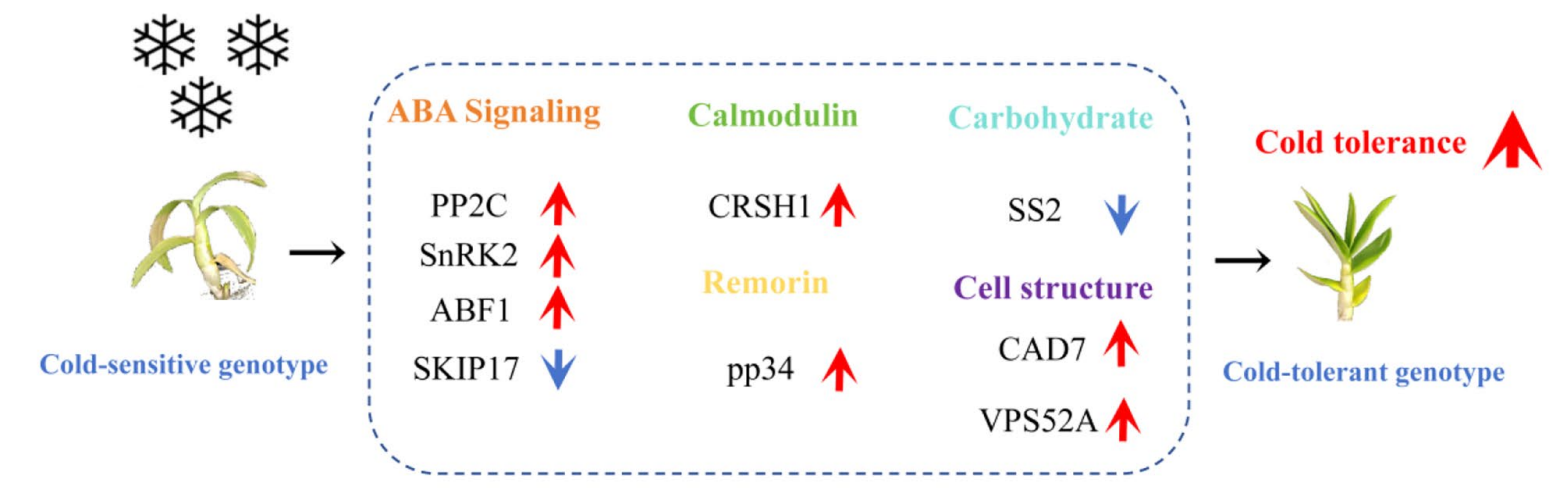
Analysis of nine hub genes responding to cold stress in *Dendrobium* spp. Analysis of gene expression patterns responsive to cold stress in *Dendrobium* spp. PP2C, protein phosphatase 2 C; SnRK2, sucrosenon-fermenting1–relatedproteinkinase 2; ABF1, ABRE binding factors 1; SKIP17, Ski-interacting protein 17; CRSH1, GTP diphosphokinase CRSH1; pp34, remorin pp34 protein; SS2, starch synthase 2; CAD7, Cinnamyl alcohol dehydrogenase 7; VPS52A, vacuolar protein sorting–associated protein 52 A. Red represents upregulated expression genes, and blue indicates downregulated expression genes

Overall, we mined and characterized *Dendrobium* spp. cold response–related hub genes that have not been fully elucidated through RNA–seq and WGCNA. Plant cold tolerance can be viewed as a quantitative trait that combines cold response–related metabolic pathways and molecular effects that mitigate cold-induced damage by regulating normal physiological metabolic activities and cellular homeostasis [63]. Moreover, we noticed that the changes of gene expression profile were faster than those of phentypic and physiological indices (Figs. 2 and 7). These phenomena were also observed in rapeseed [64] and arabidcopsis [65] under cold conditions. The changes of metabolite and phenotypes owed to multiple gene expression and biochemical cascade changes. In this study, the cold response model constructed based on co–expressed genes and hub gene expression patterns reveals *Dendrobium* spp. pathways that are activated in response to cold stress and provides guidance for cold tolerance research in other plants (Figs. 8 and 9).

## Conclusion

In conclusion, complex metabolic pathways and gene networks regulate *Dendrobium* spp. responses to cold stress. We used a combination of phenotypic, physiological, and CHM analyses to show that the physiological cold response of SH (cold–sensitive genotype) in the later stages of cold stress was similar to that of HX (cold–tolerant genotype) throughout the 16-d cold treatment. The results of the OPLS–DA analysis showed that SP and POD are the key physiological parameters for assessing *Dendrobium* spp. response to cold stress. Moreover, the metabolic pathways involved in the *Dendrobium* spp. cold stress response include signal transduction, phytohormones, carbohydrates, cell structure, among others. The WGCNA results showed that signal transduction pathways (including serine/threonine kinases, MAPK signaling, Ca^2+^ signaling, calmodulin and remorin), the ABA signaling pathway, starch and sucrose metabolism, and cell wall and endocytosis in cell structures are in the hub positions of the *Dendrobium* spp. cold response. In particular, the genes *pp34*, *PP2C*, *SnRK2*, *SKIP17*, *ABF1*, *CRSH1*, *SS2*, *CAD7*, and *VPS52A* represent major hubs in the in *Dendrobium* spp. cold response. This study provides strategies to investigate the cold tolerance mechanisms of cold–tolerant *Dendrobium* spp. and other flowering crops.

## Methods

### Experimental design and plant sample preparation

*Dendrobium* spp. seedlings were planted in the tropical orchid nursery of the Tropical Crops Genetic Resources Institute at the Chinese Academy of Tropical Agricultural Sciences. Two Chinese cultivars of *Dendrobium* spp., ‘Hongxing’ (HX, a cold–tolerant cultivar) and ‘Sonia Hiasakul’ (SH, a cold–sensitive cultivar), were subjected to cold treatment (10 °C). Seedling age and size were consistent for the two tested *Dendrobium* spp. varieties. Crude coconut shells were used as a medium for *Dendrobium* spp. cultivation. The experiments were conducted at the Sanya Institute of China Agricultural University (18°33’N, 109°18’E). Two *Dendrobium* spp. cultivars of medium seeding were placed in a climatic laboratory at 10 °C with 65% humidity and a 14-h light/10-h dark photoperiod. Leaves from the two *Dendrobium* spp. cultivars were collected at 0, 1, 2, 4, 8 and 16 d under 10℃ cold treatment. The top three leaves of *Dendrobium* spp. plants were selected for physiological indicator determination and sequencing. Each sample was set in three biological replicates, and samples were stored at − 80℃.

### Plant phenotype and electrical conductivity analysis

Plant defoliation rate and leaf yellowing rate were calculated after the cold stress treatment. Chlorophyll content was quantified as in Lu et al. [5]. Electrolyte leakage (relative electrical conductivity) was evaluated using the methods described by Li [66].

### Physicochemical analyses of leaves under cold treatment

Physiological indicators were assessed in two *Dendrobium* spp. cultivars under 10℃ cold stress. Peroxidase (POD) activity was determined using the phenol method of Luo et al. [67]. Malondialdehyde (MDA) content was quantified according to the thiobarbituric acid method, as described by Wu et al. [68]. Free proline (FP) content was analyzed using ninhydrin colorimetric analysis as reported by Patel and Vora [69]. Soluble protein (SP) and soluble sugar (SS) contents were determined using Coomassie brilliant blue [70] and the anthrasterone colorimetric method [9], respectively.

### RNA sequencing analysis

Based on the previous data and phenotypic change of *Dendrobium* spp. under cold treatments, the 0, 4 and 8 d cold samples were used to compare the transcriptional profiles [5]. *Dendrobium* spp. leaves (0.2 g) were sampled for RNA extraction according to the instructions provided in the RNA kit (Beijing Tiangen Biotechnology Co., Ltd., China). The purity and integrity of the total RNA were assessed using the Agilent Bioanalzer 2100 system (Agilent Technologies, Palo Alto, CAUS). RNA samples (25 µg; OD_260_/_280_ > 2.0) were prepared to build the sequencing library using the NEBNext® Ultra™ RNA Library Preparation Kit (NEB Next UltraTM, USA).

The constructed libraries were sequenced on the Illumina Novaseq 6000 platform (Illumina Inc., San Diego, CA, USA) to generate paired–end reads. Data assembly and mapping of the reads were performed using StringTie [71]. DESeq was used to analyze differential expression [72], and to calculate the Fragments Per Kilobase of transcript per Million mapped reads (FPKM) values. Genes whose Variable Importance in the Projection (VIP) > 1.0, false discovery rate (FDR) < 0.05, and fold change (FC) ≥ 2 were identified as significantly differentially expressed genes.

Functional annotation of *Dendrobium* spp. genes was performed using the following databases: (1) NCBI non–redundant protein (Nr); (2) the Kyoto Encyclopedia of Genes and Genomes (KEGG); (3) NCBI nonredundant nucleotide (Nt); (4) Protein family (Pfam) analyzed based on the HMMER 3.3 package; (5) Clusters of Orthologous Groups of proteins/euKaryotic Orthologous Groups (COG/KOG); (6) Gene Ontology (GO) annotations performed by Blast2GO (version 2.6.0); (7) Swiss–Prot (an annotated protein sequence database).

### Weighted gene co-expression network analysis (WGCNA) and hub gene screening

The WGCNA [73] R software package (version 3.4.1) was used to construct and analyze the co–expression network. The RNA–seq data used for the WGCNA were obtained from 18 samples (three timepoints for two cultivars) (Fig. 6A). Hierarchical clustering trees were constructed to exploit the topological overlap matrix similarity, and modules were detected with reference to the dynamic tree–cutting algorithm [74]. The power, minModuleSize, and mergeCutHeight in this network were set to 15, 100, and 0.05, respectively. Furthermore, the correlation coefficients among the hub genes in the module were determined. Genes with a kME greater than 0.7 were considered hub genes and used to represent the expression pattern of the whole module. The top 105 hub genes in the co–expression network were visualized using Cytoscape software (version 3.7.1) [75].

### Hub gene analysis by reverse transcription quantitative PCR

Reverse transcription quantitative PCR (RT-qPCR) was performed to validate the RNA sequencing (RNA–seq) data for a subset of hub genes derived from the top 105 hub genes. cDNA synthesis was performed according to the manufacturer’s instructions using PrimeScript RT Master Mix (TaKaRa, Dalian, China). The qPCR results were analyzed using a Bio–Rad CFX ligation fluorescence quantitative qPCR detection system. The *Actin* gene was used as a reference control. Primers (Table S1) designed for qPCR were obtained from Primer–BLAST of the NCBI website (https://www.ncbi.nlm.nih.gov/). Relative expression levels were obtained based on the 2^−ΔΔCT^ method described by Livak and Schmittgen [76].

### Statistical analysis

Analysis of variance (ANOVA) and Tukey’s test were performed on the physiological data, and normal distribution maps were plotted using SPSS 22.0 (IBM, Armonk, NY, USA). Origin 2018 (Origionlab, Northampton, USA) was used for PCA. A CHM analysis of the physiological indices was performed using TBtools. OPLS–DA was conducted based on the R software package (version 3.4.1).

### Electronic supplementary material

Below is the link to the electronic supplementary material.

Supplementary Material 1

Supplementary Material 2

Supplementary Material 3

Supplementary Material 4

Supplementary Material 5

Supplementary Material 6

Supplementary Material 7

## Data Availability

The raw transcriptome data have been deposited at the NCBI Sequence Read Archive under the accession number PRJNA1041885 (https://www.ncbi.nlm.nih.gov/sra/PRJNA1041885).

## Abbreviations

HX: Hongxing
SH: Sonia Hiasakul
OPLS–DA: Orthogonal partial least squares discriminant analysis
WGCNA: Weighted gene co-expression network analysis
KEGG: Kyoto Encyclopedia of Genes and Genomes
GO: Gene ontology
PP2C: PROTEIN PHOSPATASE 2 C
SnRK2: SNF1-RELATED PROTEIN KINASE 2
ABF1: ABRE-BINDING FACTOR 1
SKIP17: SKI-INTERACTING PROTEIN 17
RCH1: Ca^2+^ signaling-related GTP diphosphokinase gene
SS2: STARCH SYNTHASE 2
CAD7: CINNAMYL ALCOHOL DEHYDROGENASE
VPS52A: VACUOLAR PROTEIN SORTING-ASSOCIATED PROTEIN 52 A
COLD1: CHILLING-TOLERANCE DIVERGENCE 1
TFs: Transcription factors
CORs: Cold-regulated genes
ROS: Reactive oxygen species
MAPKs: Mitogen-activated protein kinases
CDPKs: Ca^2+^-dependent protein kinases
ICE1: INDUCER OF CBF EXPRESSION 1
ABA: Abscisic acid
CRT: C-repeat-binding factor
ABFs: ABA–responsive element-binding factors
AREBs: ABA-responsive element-binding proteins
DREB: DEHYDRATION-RESPONSIVE ELEMENT BINDING
REC: Relative electrical conductivity
POD: Peroxidase
MDA: Malonaldehyde
FP: Free proline
SP: Soluble protein
SS: Soluble sugar
CHM: Cluster heatmap
PCA: Principal component analysis
VIP: Variable Importance in the Projection
DEGs: Differentially expressed genes
FPKM: Fragments Per Kilobase of transcript per Million mapped reads
FDR: False discovery rate
CaMLs: Calmodulin–like proteins
CBLs: Calmodulin–binding proteins
PM: Plasma membrane
CME: Clathrin–mediated endocytosis
CIE: Clathrin–independent endocytosis
FC: Fold change
Nr NCBI: non–redundant protein
Nt NCBI: nonredundant nucleotide
Pfam: Protein family
COG/KOG: Clusters of Orthologous Groups of proteins/euKaryotic Orthologous Groups
RNA–seq: RNA sequencing
ANOVA: Analysis of variance
AUX1: Auxin–responsive gene
RT-qPCR: Quantitative PCR
