# Supplementary material for: Physiological and transcriptomic comparisons shed light on the cold stress response mechanisms of Dendrobium spp
Source: BMC Plant Biol. 2024 Apr 1;24:230. doi: 10.1186/s12870-024-04903-1 (PMC10985946; doi:10.1186/s12870-024-04903-1)

**Supplementary figure caption**

**Fig. S1.** The Kolmogorov-Smirnov normality test of physiological data. SD, standard deviation; N, number of samples.

**Fig. S2**. Correlation analysis and PCA analysis of the 18 *Dendrobium* spp samples. (**A**) Pearson correlation between samples; (**B**) PCA analysis between the samples.

**Fig. S3**. Co-expression network of the top 105 hub genes from the 12 module responding to cold stress. Node size represents connectivity, and node color represents category.

**Fig. S4.** Pathways of *Dendrobium* spp from KEGG. (**A**) ABA pathway (ko04075) of *Dendrobium* spp from KEGG. ABA: abscisic acid; PYR: pyrabactin resistance; PYL: PYR1–like protein; RCAR: regulatory components of ABA receptor; PP2C: type 2C protein phosphatases; SnRK2: Sucrosenon–fermenting1–relatedproteinkinases 2; ABF1: ABRE binding factors 1; (**B**) Endocytosis pathway (ko04144) of *Dendrobium* spp from KEGG.

**Fig. S1**


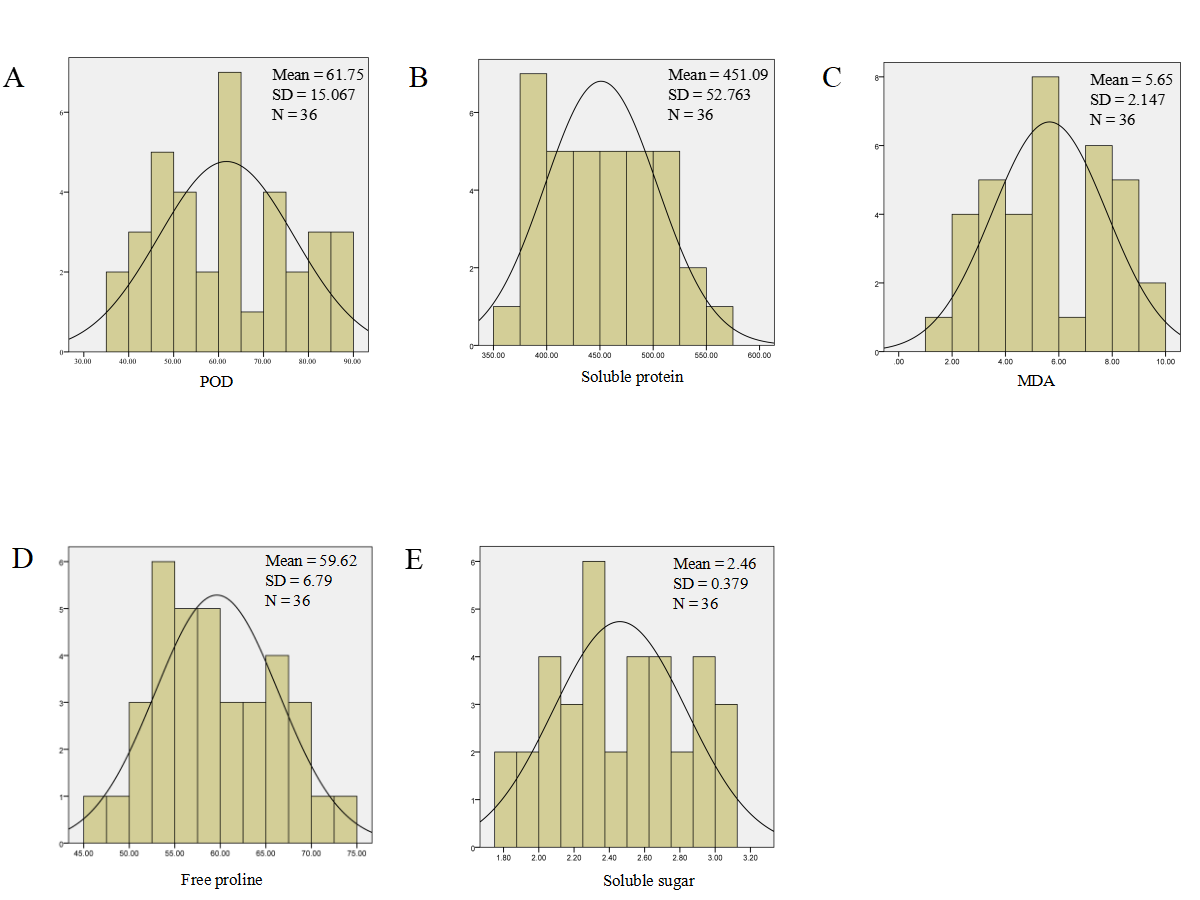


**Fig. S2**

**Fig. S3**

**Fig. S4**


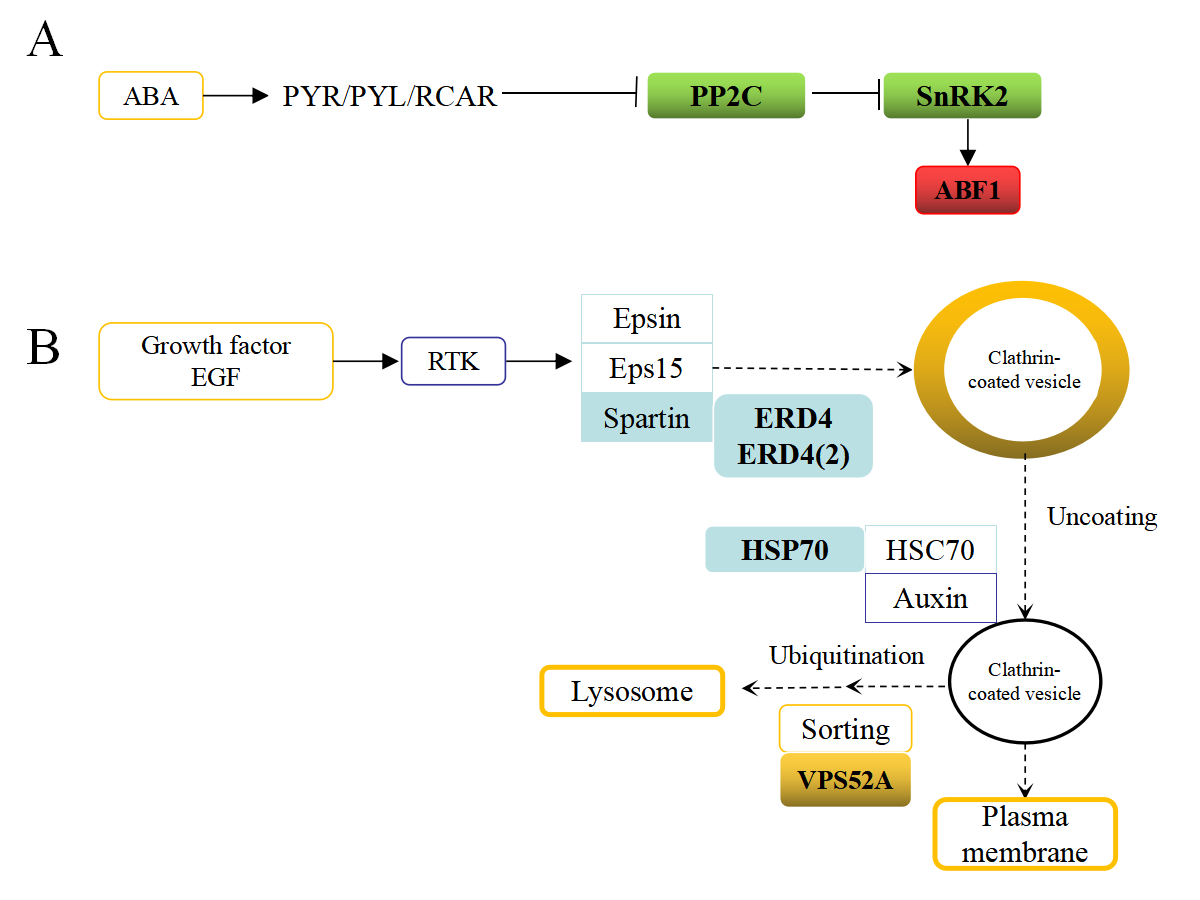

Supplement: Supplementary file 1 — Supplementary Material 1 [file 12870_2024_4903_MOESM1_ESM.docx]
